# Theoretical Considerations on the Literacy-Metacognition Nexus: Exploring the Linguistic-Cognitive Landscape of Young Multilingual Minds

**DOI:** 10.3390/brainsci14100979

**Published:** 2024-09-27

**Authors:** Barbara Hofer, Birgit Spechtenhauser

**Affiliations:** 1 Faculty of Education, Free University of Bozen-Bolzano, 39100 Bozen, Italy; barbara.hofer1@unibz.it; 2 Department of English, University of Innsbruck, 6020 Innsbruck, Austria

**Keywords:** multilingual literacy, metalinguistic awareness, (meta)cognition, executive functions, cognition-literacy-multilingualism triadic relationship, multilingual development, multilingual competence, multilingual system

## Abstract

Background/Objectives: Research suggests that metalinguistic and cognitive/attentional-control processes are key variables in literacy development in young learners. Interactions between these variables are complex, and this complexity is increased in multilingual learners. With data on the interplay between metalinguistic and cognitive awareness, literacy, and multilingualism being scarce, it is far from clear how these variables interact and how they impact the individual child. This article sets out to shine some light on the interconnectedness and interactions between metalinguistic awareness, cognitive/executive functions, and (multilingual) literacy. Conclusions: We argue that the three dimensions are strongly correlated and that this correlation comes with important implications for language learning, language processing, and language development. However, the exact nature of these correlations is yet to be established.

## 1. Introduction

**Figure 1:**
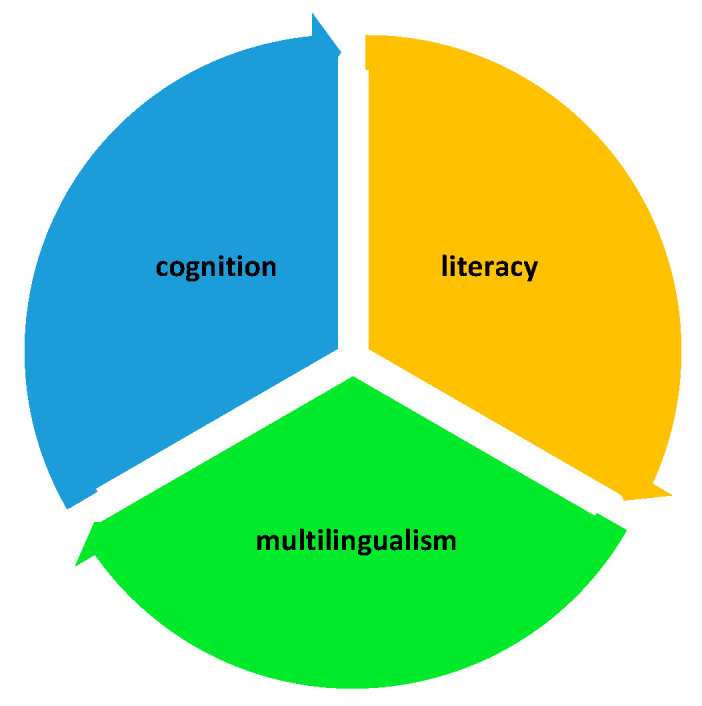
Visual representation of the cognition-literacy-multilingualism triadic relationship.

Research in neuroscience and cognitive and developmental psychology points to an important nexus between linguistic and cognitive processes and children’s literacy development. The complex dynamics and the forces that drive this interaction are at the heart of the present contribution. A psycholinguistic, systems-theoretical, multilingual perspective [1,2] informs our discussion. Particular attention is given to the interface between language (and metalinguistic awareness), cognition (executive functions), and literacy in multilingual children. The focus on multilingualism is motivated by our own research and interest in multilingual competence building [3,4] and the relationship between language, metacognition, and multilingual development [5,6]. The aim is to elucidate some of the mechanisms that underpin the interactions within this triadic relationship and explore how the three dimensions interact and mutually influence each other. We start with some conceptual and terminological clarifications and then proceed to discuss the triad language-cognition-literacy with a particular view on multilingualism and early multilingual development. Rather than conceiving of them as separate categories, each working in isolation from the other, we propose to construe them as overlapping and intersecting to a significant degree. Empirical research provides support to the effect that language is closely intertwined with meta-linguistic/meta-cognitive awareness and ability and that cognitive processes as ensconced in executive functions (including working memory, attentional control processes, inhibition and shifting) provide a strong underpinning for all forms of language processing and literacy development. The triadic representation of language, cognition, and literacy in Figure 1 below serves the purpose of modeling and visualizing the mutual relationship between the three dimensions.

The subsequent section deals with executive functions, what they are and entail and why they are relevant for our discussion of the language-cognition-literacy connex. Due to obvious limitations, we can only give a perfunctory overview. Interested readers are referred to the pertinent literature e.g., [7,8,9].

## 2. Executive Functions

Executive functions (EFs) constitute a sort of meta-cognitive supervisory or control system. They mediate human behavior and are prominently involved in learning, problem-solving, self-monitoring and self-regulation, school readiness, and academic functioning. As such, EFs provide the foundational and functional basis for language learning and processing, and we make a point of arguing for multilingual development and multilingual competence building. From this, we infer that they co-determine learning progression, learning success, and learning outcomes, as reflected in negative and positive growth patterns. The evidence here comes, for instance, from research that found that children with poorly developed EFs also do poorly on various verbal and non-verbal measures [10]. It is important to note that EFs are not only linked to academic success and school performance [11] but to success in life more generally. In fact, they impact the full range of human life. Empirical studies show that EFs are linked to socio-emotional competence [12,13] and socio-economic status [14] and that they even have a role to play in criminal behavior in adulthood [10]. Poarch and van Hell [15] (p. 3) note that the

“human cognitive system is faced daily with situations in which a choice is required between two or more alternative responses that are in competition with one another [16] (Keye, Wilhelm, Oberauer, and Van Ravenzwaaji, 2009). In such situations, our cognitive system needs to rely on conflict monitoring mechanisms that allow for conflict detection and the subsequent resolution of such conflict.”

The literature proposes three broad types of EFs, including *response inhibition, working memory,* and *cognitive flexibility,* which are all, to some extent, involved in goal-directed behavior [17] and guide attentional processes and human actions, as well as learning behavior. In very broad terms, *response inhibition* allows the child to differentiate between distracting and task-relevant information, resist prepotent stimuli, and inhibit interfering distractors. As a consequence, it allows learners to set priorities (which is key in writing tasks, self-regulatory processes, learning behavior, etc.). *Working memory* helps learners retain, monitor, manipulate, and update verbal and non-verbal information. It supports the retention of information in short-term memory and allows the individual to structure her/his utterances in such a way that they do not constantly repeat themselves but produce relevant and meaningful messages. Finally, *cognitive flexibility* relates to the capacity to shift attention in response to changing demands and to adapt to new situational (or task) requirements [10].

The point we are making is that the three EFs are also involved in language acquisition and processing cf. [18], and, more specifically, multilingual learning. Research—whether from a psycholinguistic or neuroscientific perspective—on the interrelatedness and mutual interplay of cognition, literacy, and multilingualism is, however, scarce. Indeed, to the best of our knowledge, there are barely any empirical data on how metalinguistic and metacognitive ability correlate with literacy and multilingual development. While recent studies in second and third-language acquisition and multilingualism have found important correlations between meta-linguistic/meta-cognitive awareness and bi- and multilingual competence, the triadic relationship addressed here has not to this day been explored in any depth or width. With the present contribution, we aim to shed more light on how the three dimensions interlink and to determine whether educational and diagnostic implications can be inferred from the current state of research. It is important to note, though, that while we expect the interplay between cognition-multilingualism-literacy to have a significant impact on children’s meta-cognitive and linguistic development, we do not yet fully understand the exact nature of the triadic interactions under discussion, nor for that matter, about the resulting developmental consequences for the individual child. Ours is, therefore, a first foray into uncharted territory.

### Executive Functions and Language

EFs are directly correlated with language. Correlational studies have shown that stronger EFs come with better language skills, particularly in the areas of working memory, inhibition, cognitive flexibility, and shifting foci between tasks and goals. EFs are, in this sense, supportive of meta-linguistic thinking, language processing, and language acquisition and development. Young children with strong EFs tend to have larger vocabulary and demonstrate better syntax/grammar comprehension, as well as enhanced language learning abilities [19]. Grammar learning and developing syntax knowledge involve a whole series of cognitive-executive functions, including attention, control, memory, and flexibility.

Research has also shown that attention and working memory (WM) facilitate the integration of lexical meanings and syntactic knowledge and contribute to their anchoring in short-term memory [20]. WM allows speakers to retain information when communicating orally and/or in writing or signing. Together with attentional control and a degree of mental flexibility, WM allows individuals to deploy their linguistic resources in a targeted manner. It capacitates them to modify and adapt their linguistic output so as to accommodate different interlocutors, and it makes sure that the linguistic output they generate is clear, cohesive, and pertinent [20].

Investigations contrasting typically developing children with atypical children provide further evidence of the link between EFs and language development. Numerous studies have shown that children with developmental language impairment [21] or disorders such as autism [22] or dyslexia [23] have weaker EF skills compared to normally developing children. However, while the relationship between EFs and language is well established, existing research has not been able to clearly determine the direction and strength of these relationships (i.e., whether EFs influence language in the same way as language impacts EFs). So far, the few studies that have specifically investigated the directionalities between EFs and language development have yielded mixed results.

## 3. Metalinguistic Awareness (MeLA), EFs and Early Literacy Acquisition

Conceptualized as “a construct midway between language and metacognitive abilities” [24] (p. 12), metalinguistic awareness and ability (henceforth MeLA) relate to the child’s understanding of language forms (e.g., lexical, phonological, grammatical, morphosyntactic) and functions (i.e., how language is or can be used and manipulated). MeLA enables the child to think about and reflect on language in all its diverse manifestations and to strategically deploy language for their own purposes. MeLA entails knowledge about language and the ability to *reflect and act upon* language as an arbitrary system of rules, conventions, and meanings. In very young learners (up to around age 5), language awareness tends to emerge in embryonic forms at a somewhat intuitive, *epilinguistic* level [24]. In older children, awareness is *metalinguistic*, which is to say that it takes effect at a meta-level, viz., at a level of high(er) abstraction and elaboration. At this elevated level, MeLA involves higher-order cognitive processing and skill and can (to varying degrees of sophistication and explicitness) be externalized through verbal reports. While MeLA develops in monolingual, bilingual, and multilingual speakers, it is important to note that the nature of metalinguistic understanding, metalinguistic thinking, and metalinguistic skill changes significantly from mono- to bi- and multilinguals (as we explicate below).

MeLA plays a central role in literacy development [25,26]. Children who early develop a‚ metalinguistic mind [24] (p. 12) have a better grasp of how language works. They approach language in a more systematic and analytical way compared to children with poorly developed MeLA, and they have a better understanding of lexico-semantic and morpho-syntactic categories, as well as pragmatic aspects of language, which are all key in literacy and narrative competence development [27]. Reading and writing, in particular, benefit from high levels of MeLA [28]. Reading and writing presuppose good planning, organizing, and structuring of language and text (whether oral, written, signed, multimodal, or other). They require revising and editing and, by implication, the ability to rephrase, restructure, and rearrange elements in text. In very young learners, MeLA (mediated by phonological awareness and a preliminary understanding of phoneme-grapheme correspondence, letters, words, and/or grammatical categories as constitutive of printed text) scaffolds first reading attempts. There is broad agreement that EFs—notably, attention focusing and inhibitory control, working memory, and self-regulation—have a similarly important role to play in reading. The understanding is that EFs ease the cognitive load when children are engaged in synthesizing and blending sounds together. They mediate processes of integration and interpretation of grapho-phonological, semantic, and syntactic information, and they mediate inhibition of distractions deriving from noises in the environment or within (e.g., when children are tired or lack motivation and/or self-regulation to keep going), etc. Together then, MeLA and EFs help children overcome cognitive conflict, which they encounter when decoding and making sense of what they are reading, and they help them persist in the reading task. MeLA and EFs also team up when it comes to writing. MeLA and EFs are critical determinants at all stages of text production and narrative competence, both of which presuppose abilities such as organizing and arranging informational content in a coherent and cohesive way so that readers can follow the author’s train of thought and their line of argumentation. Bialystok and Barac [29] (p. 71) argue that metalinguistic skill improves with increased knowledge of the language, while executive control performance benefits from increased experience in bilingual educational environments, i.e., literacy instruction. Cognitive development, they state, “proceeds as children build more structured representations of knowledge and gain greater control over attentional procedures” [29] (p. 72). The representational structure is mediated by an increase in knowledge, and metalinguistic ability is very much dependent on linguistic representation (viz., knowledge structures). Control, by contrast, seems to be more sensitive to experience and practice cf. [29]. Recent studies indicate that EFs and metacognition follow similar developmental trajectories, with each improving consistently from early childhood through adolescence [30,31].

## 4. The Multilingual Advantage

There is growing (though by no means universal)^1^ consensus that learners/users of multiple languages benefit from a multilingual advantage^2^ when carrying out complex, cognitively challenging tasks [32] (p. 3)^3^. Complex tasks can include anything from problem-solving and conflict resolution to language and literacy learning. The DMM [1,2] posits a so-called M(ultilingualism)-effect which emerges in users of multiple languages due to the presence and interaction of multiple language systems in the mind cf. [34]. The M-effect comes with an enhanced metalinguistic and metacognitive skillset [35], high levels of cognitive flexibility (i.e., elasticity and plasticity), new systems properties inclusive of an enhanced monitor, and strong language management, language learning, and language maintenance skills cf. [2,4,6]. The understanding is that multilingualism can enhance (meta)cognitive control and EFs, strengthen learners’ self-monitoring and self-regulation capacities, their metalinguistic awareness and abilities, and by implication, their literacy development, which, as explicated, relies on higher-order cognitive and metacognitive skills and is assisted by metalinguistic knowledge and ability. Bialystok [34] assumes such a training effect in bilinguals cf. [36]. Bilingualism, she states, “trains” EFs through its constant recruitment for language selection. Referring to the extant literature, Ware et al. [37] (p. 1) similarly note that bilingualism is linked to better performance in executive control and changes in brain structure and function. Whether this also applies to multilinguals has not to this day been extensively studied (but see Schroeder and Marian [36] (p. 760) for some preliminary evidence to this effect; see also findings discussed in Section 6). Nor is it clear who, if there is such a training effect, counts as multilingual. Is it only children who grow up with more than two languages, or is it anybody who acquires their languages later in life, and what about proficiency? Do children, as Cummins [38] implies, need to reach a minimum level of proficiency for any benefits to obtain, and if so, how do we establish any such benchmark? In the following, we provide the first CDST-informed answers to some of these questions.

## 5. Becoming Multilingual and Multi-Literate

This section aims to clarify what we mean by multilingualism and multiliteracy. Much of the discussion around multiliteracy centers on proficiency and native speaker target norms. Narrow views, according to which only balanced bi- or multilinguals, who come close to the idealized native speaker competence in each of their languages, count as bi- or multilingual, have long dominated the scholarly debate on this matter. However, there is widespread consensus today that such a limiting perspective does not accurately represent the way multilinguals learn and orchestrate the various languages they have in their minds. Consequently, in the current discussion, the perfect mastery of each of the multilingual’s languages is no longer regarded as an essential requirement to be considered multilingual [39,40]. Grosjean [41] and Cook [42,43], who laid the groundwork for this perspective, emphasize that a bilingual is not a ‘double monolingual’ (and, by extension, a multilingual is not merely a ‘multiple monolingual’). Instead, multilinguals are seen as having unique linguistic constellations in their minds, making any measurement of their skills based on the so-called ‘monolingual yardstick’ or ‘monolingual norm’ [1,40,44]—i.e., comparing the competence of each language in a multilingual’s repertoire to that of a monolingual—ineffectual and inadequate A complex dynamic systems perspective compels us to look at the whole picture including a whole range of variables (such as communicative needs, social contexts, professional demands, attitudes towards language learning, etc.). and to conceptualize multilingualism in terms of continua rather than in terms of categories. From this perspective, the multilingual system can then be understood as a system in constant flux [1,2], which evolves throughout an individual’s life and is, in this sense, a kind of mirror image of their psycho-social trajectory cf. [45]. From this, it follows that individuals can become multilingual at different stages in their lives because (multilingual) systems are capable of continuous change and adaptation to new circumstances.

To grasp the complexity of multilingual development, it is essential to examine the language-cognition-literacy construct in the multilingual mind from a holistic, multi-competence perspective cf. [4,6,40]. What is crucial in this process is that we find ways of measuring multilingual competences and multiliteracy more specifically that extend beyond the elicitation of isolated and exclusively language-specific aspects (which are benchmarked against monolingual norms and also include metacognitive dimensions of language use in an effort to capture multilinguals’ linguistic-cognitive constellation as fully as possible. This holistic approach can then serve as a foundation for a more in-depth exploration of literacy development across various languages.

## 6. Multi-Literacy, EFs, and MeLA Development

The existing literature, as we pointed out previously, indicates significant correlations between reading and writing performance EFs and MeLA, but also see [46,47]. However, most studies focus on isolated EFs or MeLA aspects (e.g., working memory, episodic memory, cognitive inhibition, morphological awareness, phonological awareness) and often investigate monolingual or, at best, bilingual individuals. Nevertheless, a few studies address the role of EFs and/or MeLA among bilinguals when learning to read and write in a third language. For example, Rauch et al. [48] examined the influence of biliteracy on reading proficiency in a third language and the involvement of MeLA in this process. Still, there remains a small body of research that focuses on more comprehensive cognitive strategies used by multiple language learners (with more than two languages in their repertoire) when learning a new language, particularly in terms of reading and writing, and the role of EFs and/or MeLA in this context. For instance, Dahm [49] investigated the multilingual cognitive advantage, as discussed in Section 4, and addressed training effects on transferable learning strategies that include cognitive strategies. In her 2015 study, Dahm [49] trained French students to use various learning strategies to facilitate language learning and to transfer these strategies to new learning situations (including reading and writing activities). This approach incorporated didactic concepts that involve multiple languages and draws on the Pluralistic Approaches based upon Unknown Languages (PAUL), as described by Candelier [50]. In her investigation, participants were tasked with solving the problem of accessing meaning in three short texts written in unknown languages. Dahm [49] found training effects and concluded that explicit declarative MeLA is essential to effectively transfer cognitive (learning) strategies. Similarly, Spechtenhauser and Jessner [6] explored multilingual strategies and, in particular, the metacognitive strategies used by young multiple language learners with different MeLA competences (assessed through trilingual MeLA tests) during decoding processes as well as the levels of awareness involved in these processes, also highlight the importance of metalinguistic awareness for successful decoding. In their study, in which trilingual South Tyrolean students were required to decode a comic in French—a language system the participants were not familiar with—the findings revealed that especially when participants could activate the highest level of awareness (see also Leow [51] for levels of awareness) and explicitly verbalize their metalinguistic thinking, decoding at the level of understanding could be achieved. Notably, those who attained high MeLA scores on the MeLA tests were particularly adept at activating the highest level of awareness when decoding the new linguistic system, which in turn led to more effective decoding. Similar effects of cross-linguistic awareness were found in Hofer’s [4] research with children in German and Italian-medium primary schools. A long-term effect that can be achieved through explicit training of metalinguistic and specifically cross-linguistic awareness over an entire school year was demonstrated in a study carried out within the Austrian school context by Allgäuer-Hackl [35]. In her study, which involved comparing the performance of participants on a series of linguistic and metalinguistic tasks (primarily reading and writing tasks) with that of a control group, she was able to demonstrate a significant effect of multilingual training. The experimental group notably outperformed the control group in activities involving meta- and cross-linguistic skills (for an in-depth discussion on the role of meta- and cross-linguistic awareness in multilingually diverse learning settings, see Jessner and Allgäuer-Hackl [52]). Similarly, Forbes [53] found in her study on the development of transferable metacognitive writing strategies that explicit training of metacognitive writing strategies within the foreign language learning classroom not only enhanced learners’ performance on writing tasks in their L2 or L3 but also had a positive impact on their L1 writing skills.

These investigations highlight, as discussed in Cummins’ Iceberg Model of Language Interdependence [38], that acquired linguistic, metalinguistic abilities and literacy resources in one language can be applied when learning a new language (see Kecskes and Papp [54] for a similar conceptualization of transfer). In his Interdependence Model, Cummins [38] specifically underlines the metacognitive dimension that is involved in cognitively more demanding (academic) tasks (such as reading and writing in schooling settings), which can be transferred across languages and have a boosting effect on language learning. Cummins [38] suggests that a minimum threshold level of proficiency has to be attained so that beneficial cognitive aspects of bilingualism can come to light. He proposes two levels of threshold—the higher threshold level (a high level of competence in two languages is achieved) and the lower threshold level (a high level of competence in one language is achieved). Some scholars also argue that relatively low levels of proficiency, reached after just one or two years of structured learning, can be sufficient to show the effects of the transfer of (meta)cognitive strategies [49,55]. Hofer [4] and Spechtenhauser and Jessner [6], for instance, found in their investigations that young and adolescent learners with still quite low proficiency levels in L2 and L3 can already draw on their multilingual resources and strategically use their meta- and cross-linguistic abilities. For obvious reasons, as outlined in the DMM [2], the discussion becomes even more complex if more than two languages are involved. Lasagabaster [56], as one of the first scholars, extended Cummins’ threshold hypothesis to a trilingual situation. In his investigation with young trilingual learners in the Basque Country he tested Basque, Spanish and English proficiency as well as MeLA competences (to test cognitive effects) and found that when the two parameters (i.e., high threshold level—high competence in three languages and low threshold level-high competence in one or two of the languages) were maintained, the first group (which included those who were highly competent in three languages) performed significantly better on the MeLA test than the other group (which included those who were highly competent in one or two languages). However, if three thresholds (higher-medium-low thresholds) were established, those who were highly competent in three languages (higher threshold group) outperformed the other two groups, but there was no significant difference between those who were highly competent in two languages (medium threshold group) and those who were highly competent in one language (low threshold group). It becomes evident from such findings that the parameters used to measure linguistic proficiency and determine thresholds play a significant role when measuring the competences of multiple language users and, by implication, when investigating the triadic relationship between literacy, metacognition, and multilingualism. Although there is no universal agreement on how levels of threshold should be set, recent research, as noted above, points to notable transfer effects and metacognitive benefits already among quite young learners. However, as Lasagabaster [56] also observed in his research, variables such as socioeconomic (SES) factors and intelligence can impact linguistic-cognitive development. Family SES factors have, in fact, been identified as an important additional influencing variable within dynamic multilingual systems and can, as demonstrated by Spechtenhauser and Jessner [33] in their recent investigation, significantly impact multiple language learners’ metacognitive development, affecting both their meta- and crosslinguistic thinking. Lechner and Siemund [57], in their study, revisited Cummin’s Threshold Hypothesis [38] with trilinguals and also identified language external factors influencing literacy attainment. Their research examined the effects of bilingualism and multi-literacy on additional language acquisition and literacy development. They found that bilingual participants with strong academic literacy skills (in German and their heritage languages) are more likely to achieve better outcomes in academic English. This result supports Cummin’s Threshold Hypothesis and the possibility of meta- and crosslinguistic transfer. The findings also suggest the existence of individual thresholds, with some participants appearing to access and transfer literacy resources at lower levels of proficiency than others. The authors found that these lower-level resources are apparently influenced by SES factors. Participants who scored lower in tasks in their background languages but still achieved high literacy scores in the English task typically came from higher socioeconomic and educational backgrounds.

## 7. A Final Caveat

In this paper, there is a strong focus on the constructs of (meta)cognition, MeLA, and EFs and how they relate to literacy and multilingualism. We do not, however, wish to give the impression that we are overlooking the myriad of additional endogenous and exogenous conditional variables tied up with multilingual cognitive processes and multi-literacy development (see Section 6). Given the current space constraints, we also direct the interested reader to the Complexity Framework of Multilingual Competence for a detailed discussion of how these factors interact and influence multilingual competence (including literacy) development [4].

## 8. Conclusions

This paper deals with the interconnectedness of *literacy, metacognition, and multilingualism*. It seeks to disentangle the complex growth patterns that emerge in young multilingual learner-users who need to function in several languages. The current state of research shows that relationships between some of the factors of this triadic interplay and their implications for multiple language learning and multilingual development are apparent, but some effects are still unclear or remain unconsidered. Moreover, as aforementioned, our efforts to shine more light on these interactions also run up against several limits, which include a lack of shared terminology, different conceptual interpretations, dearth of research, absence of methodological consensus, scarcity of measuring instruments, and high complexity of the matter under scrutiny, to name but some. Consequently, unless we have more consistent and robust data on the relationship between *literacy, metacognition,* and *multilingualism* it will remain difficult to make any strong claims about their impact on multiple language learning/multilingual development, and we will not be able to make sound research-grounded recommendations for educators, the teaching profession and/or clinicians. It is to be hoped, therefore, that future research takes a more proactive interest in the triadic correlations discussed in this paper. Research should, in particular, investigate how linguistic and (meta)cognitive factors influence multi-literacy in young learners with different so-called initial conditions (e.g., linguistic experience, proficiency, and typical and atypical developmental trajectories) and, reversely, how literacy development across various languages impacts cognitive and metacognitive functions. Advancing knowledge in this field will not only benefit individual children but societies at large.

## Data Availability

Data sharing is not applicable to this article as no new data were created or analyzed in this study.

## Notes

1. See, for instance, Morton who decries an “insufferable mixture of excessive claims and weak evidence” [32] (p. 929).

2. Note that the more recent literature differentiates between bilingual and multilingual learner/users emphasizing the distinct nature, constitutiveness, and quality of the multilingual mind.

3. As explicated elsewhere, the multilingual advantage may fail to be obtained if living and/or learning conditions are unfavorable or hostile as in the case of socially or economically highly disadvantaged children who may not have access to formal education and/or grow up in contexts of neglect. The relevance of socioeconomic status (SES) factors in relation to MeLA was demonstrated in a recent study in the multilingual region of South Tyrol, which revealed significant correlations between the metacognitive development of multiple language learners and different measures of family SES [33]. (see also Section 6 on levels of thresholds and SES factors).
